# Japanese nationwide questionnaire survey on delayed cerebral infarction due to vasospasm after subarachnoid hemorrhage

**DOI:** 10.3389/fneur.2023.1296995

**Published:** 2023-11-02

**Authors:** Yusuke Nishikawa, Shigeki Yamada, Mitsuru Uchida, Tomoyasu Yamanaka, Yuki Hayashi, Hiroyuki Katano, Motoki Tanikawa, Toru Iwama, Koji Iihara, Motohiro Morioka, Mitsuhito Mase

**Affiliations:** ^1^Department of Neurosurgery, Nagoya City University Graduate School of Medical Science, Nagoya, Japan; ^2^Interfaculty Initiative in Information Studies, Institute of Industrial Science, The University of Tokyo, Tokyo, Japan; ^3^Department of Neurosurgery, Gifu University Graduate School of Medicine, Gifu, Japan; ^4^National Cerebral and Cardiovascular Center Hospital, Suita, Japan; ^5^Department of Neurosurgery, Kurume University, Kurume, Japan

**Keywords:** cerebral vasospasm, subarachnoid hemorrhage, aneurysmal, cilostazol, survey, delayed cerebral infarction, coil embolization

## Abstract

**Background and purpose:**

Various prophylactic drugs for cerebral vasospasm and delayed cerebral infarction (DCI) after subarachnoid hemorrhage (SAH) have been used in Japan. To investigate the treatment trends for cerebral vasospasm and frequency of DCI after SAH throughout Japan in 2021.

**Methods:**

In 2021 we conducted an anonymous questionnaire survey on management for preventing cerebral vasospasm after aneurysmal SAH, and the frequency of DCI. The questionnaire was emailed to 955 certified neurosurgeons at 553 hospitals in Japan. Of them, 162 hospitals (29% response rate) responded to the questionnaire. Of these, 158 were included in this study, while four hospitals that responded insufficiently were excluded. The efficacy of treatments for reducing DCI were examined through a logistic regression analysis.

**Results:**

Among 3,093 patients treated with aneurysmal SAH, 281 patients (9.1%) were diagnosed with DCI related to cerebral vasospasm. Coil embolization had significantly lower DCI frequency (6.9%), compared to microsurgical clipping (11.8%, odds ratio, 0.90; 95% confidential intervals, 0.84–0.96; *P*, 0.007). In addition, cilostazol administration was associated with significantly lower DCI frequency (0.48; 0.27–0.82; 0.026). The efficacy of cilostazol in reducing DCI remained unchanged after adjustment for covariates. The most effective combination of multiple prophylactic drugs in reducing DCI related to cerebral vasospasm was cilostazol, fasudil, and statin (0.38; 0.22–0.67; 0.005).

**Conclusions:**

This study elucidated the trends in prophylactic drugs to prevent cerebral vasospasm and frequency of DCI after aneurysmal SAH in Japan. Coil embolization and cilostazol administration showed effectiveness in reducing DCI related to cerebral vasospasm in 2021.

## 1. Introduction

Delayed cerebral infarction (DCI) associated with cerebral vasospasm is a major cause of disability or death after subarachnoid hemorrhage (SAH) (1, 2). Therefore, various prophylactic drugs for preventing DCI-related vasospasm have been used worldwide. Recently, the American Heart Association/American Stroke Association revised guidelines for the management of patients with aneurysmal SAH in 2023 (3). In this guidelines, cerebral vasospasm and DCI after SAH are as follows: early initiation of enteral nimodipine, a dihydropyridine calcium channel antagonist blocking the flux of extracellular calcium via voltage-gated calcium channels, is beneficial in preventing delayed cerebral ischemia and improving functional outcomes after aneurysmal SAH (class 1, strong; level of evidence, A), routine use of statin therapy and intravenous magnesium is not recommended (class 3, not benefit; level of evidence, A) (3). On the other hand, the Japanese guidelines for the management of stroke proposed by the Japan Stroke Society in 2021 recommended fasudil, ozagrel, cilostazol, edaravon, statin, and nicardipine as prophylactic drugs for preventing DCI-related vasospasm (4, 5), but there is no unified standards protocol. In 2022, clazosentan, a selective endothelin receptor antagonist, began to be insured in Japan for treatment of cerebral vasospasm (6–9), marking a major potential turning point in treatment against DCI-related vasospasm, since prophylactic drugs for preventing DCI-related vasospasm may begin to be integrated with treatment following the approval of clazosentan. However, no survey has been conducted on the management of cerebral vasospasm and/or DCI after SAH, nor have there been any studies on which prophylactic drugs are preferred throughout Japan, or whether the incidence of DCI varies according to region. Among the neurosurgeons certificated by the Japanese Neurosurgical Society, if their specialty was the cerebrovascular neurosurgery, they were additionally certificated as technical supervisors or specialists by the Japanese Society on Surgery for Cerebral Stroke. In addition, Japanese neurosurgeons generally perform not only microsurgery (clipping) but also endovascular surgery (coil embolization), and are responsible for the management of SAH patients from admission to discharge, including preventive treatment for cerebral vasospasm and DCI. Therefore, we aimed to investigate nationwide trends in prophylactic drugs and the incidence of DCI in Japan during the year 2021, using a questionnaire survey to hospitals with neurosurgeons certified by the Japanese Society on Surgery for Cerebral Stroke.

## 2. Material and methods

### 2.1. Methods for the nationwide epidemiological survey

In November 2022, we conducted an anonymous questionnaire survey to certified cerebrovascular neurosurgeons about treatment methods for preventing DCI-related cerebral vasospasm after aneurysmal SAH and the incidence of DCI from January 1, 2021 to December 31, 2021. As of September 2022, 7,928 neurosurgeons certified by the Japan Neurosurgical Society were engaged in medical practice. Among the 99 core facilities and 884 affiliated facilities that are eligible for specialty training programs approved by the Japan Neurosurgical Society, 553 facilities had 955 neurosurgeons (12%) including 665 technical supervisors and 290 technical certifiers of the Japanese Society on Surgery for Cerebral Stroke. The list of hospitals and neurosurgeons were available in an electronic database made available by the Japanese Society on Surgery for Cerebral Stroke. In addition, the total number of stroke patients treated at these hospitals during 2021 was obligated to be registered in the Japan Stroke Society Annual Survey of Clinical Practice in spring 2022. To avoid the need for respondents to extract data from the patients' medical records in order to answer this survey, we took care to ensure that respondents can answer the questionnaire simply by using the data registered in the annual survey. If more than one member in a single hospital was to be surveyed, only one representative member was asked to complete the Google Form (the survey is attached as a Supplementary material). Of them, 162 hospitals (response rate: 29.3 %) responded the questionnaire, but responses from four hospitals were excluded from this study because data on the incidence of DCI was missing.

### 2.2. Study population extraction

Finally, 3,093 patients in 158 hospitals were included in this study. The Ethics Review Committee determined that approval of this study by the Ethics Review Committee was not required, because this survey collected only the number of patients who received the targeted treatment and that of patients diagnosed with DCI at each hospital; no data related to patients' personal information including name, hospital ID, age, and gender were collected. Therefore, we collected the total number of cases in 2021 at the responding hospitals, as well as treatments and outcomes without omission, did not collect individual patient details, including the severity of SAH. Furthermore, to avoid the risk of identifying patients, we did not collect the names of the hospitals that responded, only the names of the regions where the hospital were located.

To confirm the presence of cerebral vasospasm, 145 hospitals (89.5%) used magnetic resonance angiography (MRA), 98 (60.5%) used computed tomography angiography (CTA), 76 (46.9%) used digital subtraction angiography (DSA), and <15% used transcranial doppler, CT perfusion, MR perfusion, and SPECT (multiple answers allowed). Moreover, DCI used in this study was defined as the development of a new lesion consistent with cerebral infarction on CT or MRI, and did not include symptomatic cerebral ischemia. We asked about the use of nine prophylactic drugs for preventing DCI-related cerebral vasospasm, as follow; fasudil hydrochloride, ozagrel sodium, cilostazol, edaravone, statins, steroids, nicardipine (not for antihypertensive), clazosentan, and eicosapentaenoic acid (EPA). Clazosentan was used in seven hospitals for clinical trials in 2021.

### 2.3. Classification of hospitals

Hospitals were classified by the coiling ratio, that was defined as the ratio of the number of aneurysmal SAH treated with coils divided by the total number of SAH, as follows: clipping-first hospitals were less than the first quartile point of the coiling ratio (<33.3%), coiling-first hospitals were greater than the third quartile point (72.5 ≤ %), and both-choice hospitals were between the first and third quartiles (33.3 to <72.5%). In addition, hospitals were classified into two groups based on the median number of SAHs, 15. Furthermore, hospitals were classified into two groups with the number of clipping and coil embolization in the third quartile, 12 and 16, respectively.

### 2.4. Statistical analysis

Mean values and standard deviations (SD) for continuous variables among more than two groups were compared using the Kruskal-Wallis test, and the Chi-square test was used to compare the proportions or categorical variables among groups. The logistic regression technique was used to generate odds ratios (ORs) and 95% confidence intervals (CIs) for the incidence of DCI. To assess the effects of modification and interaction between the prophylactic drugs for preventing DCI-related cerebral vasospasm and incidence of DCI, we conducted logistic regression analyses after adjustment for the number of SAH treatments and the treatment modality. Statistical significance was assumed at a probability (P) value of <0.05. All missing variables were considered as deficit data, and no other variables were adjusted. R software (version 4.2.3, R Foundation for Statistical Computing, Vienna, Austria, http://www.R-project.org) was used for all statistical analyses.

## 3. Results

### 3.1. Comparison of regional differences

**Figure 1:**
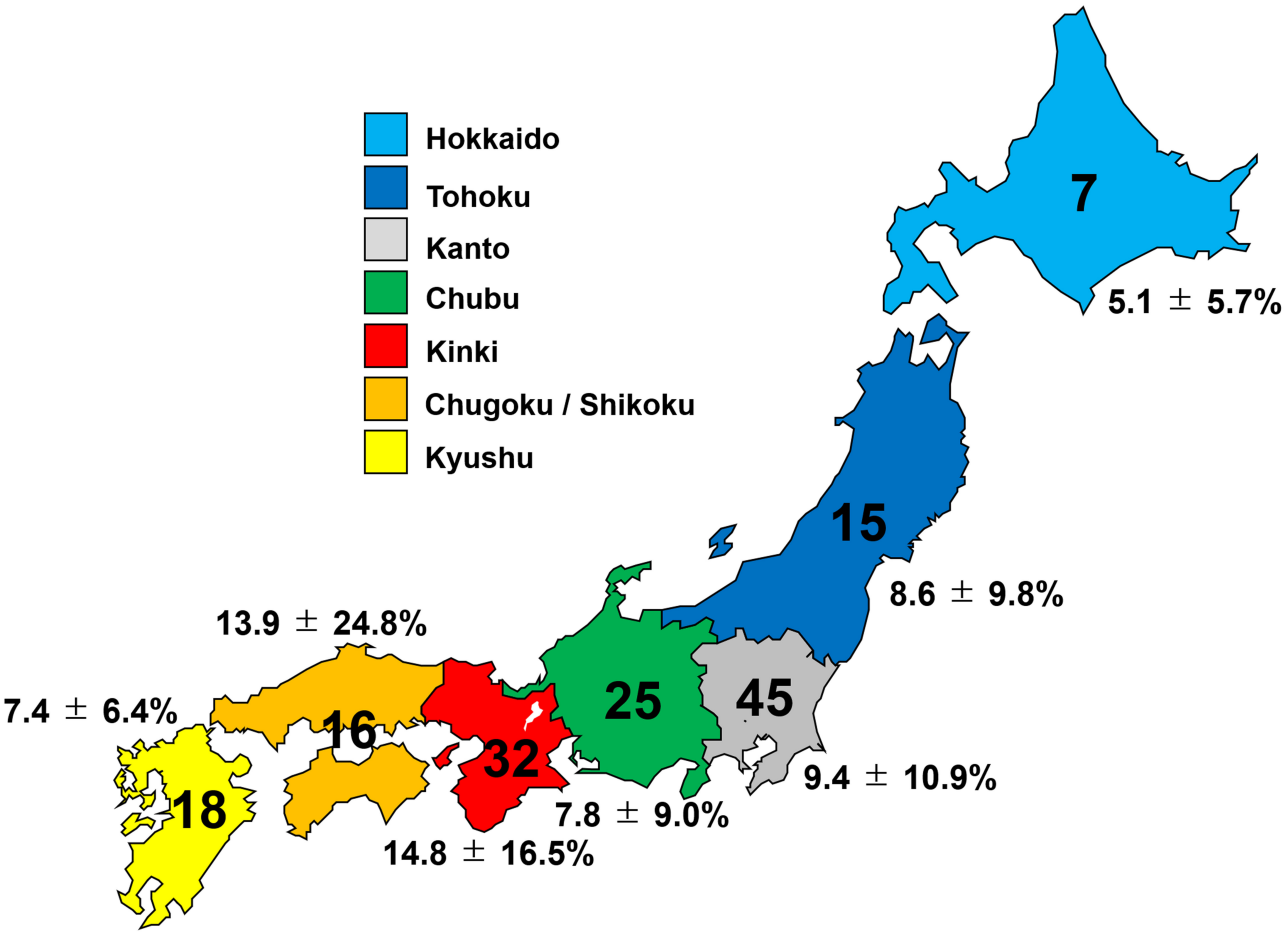
Number of hospitals participating in the study and incidence of delayed cerebral infarction (DCI) in each region of Japan.

In the nationwide questionnaire survey on the usage of prophylactic drugs for preventing DCI-related cerebral vasospasm and incidence of DCI during the year 2021, responses were received from 158 hospitals (response rate: 29.3 %). A total of 158 hospitals, and 3,093 patients who underwent the treatment for aneurysm ruptured SAH within 72 h after onset (1,401 patients treated with craniotomy and clipping, and 1,692 treated with endovascular coiling) were included. The clinical characteristics of cases in each region of Japan registered in the survey are summarized in Figure 1 and Table 1. In total, 281 patients suffered DCI after SAH among 3,093 patients (9.1%). Compared to clipping with craniotomy (165/1,401 patients, 11.8%), coil embolization had significantly lower incidence of DCI related to cerebral vasospasm (116/1,692 patients, 6.9%) (OR, 0.90; 95%CIs, 0.84–0.96; *P*, 0.007). Among Japanese geographic regions, the incidence of DCI was higher in the Kinki and Chugoku/Shikoku regions, but the difference was not significant (Figure 1, Table 1). In these regions, the DCI frequency after coil embolization was not high, but after microsurgical clipping with craniotomy was notably higher (17.1% in the Kinki region, and 21.5% in the Chugoku/Shikoku region) than in other regions.

**Table 1:** Clinical characteristics of cases in each region of Japan.

	**Total**	**Hokkaido**	**Tohoku**	**Kanto**	**Chubu**	**Kinki**	**Chugoku/ Shikoku**	**Kyushu**	***P***
Hospitals, No.	158	7	15	45	25	32	16	18	
SAHs, No.	3,093	111	430	930	442	486	311	383	0.269
Clipping, No.	1,401	89	168	450	176	216	130	172	0.301
Coiling, No.	1,692	22	262	480	266	270	181	211	0.090
DCI/SAHs, No. (%)	281 (9.1)	7 (6.3)	29 (6.7)	80 (8.6)	32 (7.2)	68 (14)	39 (12.5)	26 (6.8)	0.511
DCI/SAHs treated by clipping, No. (%)	165 (11.8)	4 (4.5)	15 (8.9)	49 (10.9)	18 (10.2)	37 (17.1)	28 (21.5)	14 (8.1)	0.617
DCI/SAHs treated by coiling	116 (6.9)	3 (13.6)	14 (5.3)	31 (6.5)	14 (5.3)	31 (11.5)	11 (6.1)	12 (5.7)	0.638
SAHs, mean ± SD	19.6 ± 15.1	15.9 ± 15.8	28.7 ± 25.6	20.7 ± 13.6	17.7 ± 11.2	15.2 ± 10.1	19.4 ± 12.0	19.4 ± 21.3	0.270
Coiling, mean ± SD	10.7 ± 10.3	3.1 ± 3.4	17.5 ± 17.0	10.7 ± 9.3	10.6 ± 7.9	8.4 ± 8.2	11.3 ± 10.5	11.5 ± 11.7	0.089
Clipping, mean ± SD	8.9 ± 8.4	12.7 ± 13.1	11.2 ± 12.1	10.0 ± 7.8	7.0 ± 6.5	6.8 ± 5.3	8.1 ± 6.0	11.6 ± 9.6	0.300
Coiling/SAHs, mean ± SD	51.9 ± 26.5	29.2 ± 29.0	52.8 ± 25.5	50.2 ± 23.1	60.6 ± 22.1	51.4 ± 29.0	54.8 ± 31.2	28.9 ± 50.5	0.360
DCI, mean ± SD	1.8 ± 2.0	1.0 ± 1.2	1.9 ± 1.9	1.8 ± 2.0	1.3 ± 1.5	2.1 ± 2.2	2.4 ± 2.7	2.3 ± 1.4	0.510
DCI/SAHs, mean ± SD	10.1 ± 13.5	5.1 ± 5.7	8.6 ± 9.8	9.4 ± 10.9	7.8 ± 9.0	14.8 ± 16.5	13.9 ± 24.8	7.4 ± 6.4	0.430
DCI after clipping, mean ± SD	1.0 ± 1.5	0.6 ± 0.8	1.0 ± 1.3	1.1 ± 1.4	0.7 ± 0.9	1.2 ± 1.7	1.8 ± 2.3	1.4 ± 0.8	0.620
DCI after coiling, mean ± SD	0.7 ± 1.0	0.4 ± 0.8	0.9 ± 1.4	0.7 ± 1.0	0.6 ± 0.9	1.0 ± 1.1	0.7 ± 1.0	1.0 ± 0.7	0.640
Hospitals with < 15 SAHs, No. (%)	72 (45.6)	4 (57.1)	4 (26.7)	17 (37.8)	11 (44)	18 (56.3)	8 (50)	10 (55.6)	0.425
Hospitals with ≤ 15 SAHs, No. (%)	86 (54.4)	3 (42.9)	11 (73.3)	28 (62.2)	14 (56)	14 (43.8)	8 (50)	8 (44.4)	
Hospitals with < 16 coiling, No. (%)	78 (49.4)	6 (85.7)	4 (26.7)	23 (51.1)	10 (40)	20 (62.5)	6 (37.5)	9 (50)	0.094
Hospitals with ≤ 16 coiling, No. (%)	80 (50.6)	1 (14.3)	11 (73.3)	22 (48.9)	15 (60)	12 (37.5)	10 (62.5)	9 (50)	
Hospitals with < 12 clipping, No. (%)	78 (49.4)	3 (42.9)	7 (46.7)	17 (37.8)	16 (64)	20 (62.5)	5 (31.3)	10 (55.6)	0.157
Hospitasl with 12 ≤ clipping, No. (%)	80 (50.6)	4 (57.1)	8 (53.3)	28 (62.2)	9 (36)	12 (37.5)	11 (68.8)	8 (44.4)	
Clipping-first hospitals, No. (%)	37 (23.4)	4 (57.1)	3 (20)	10 (22.2)	4 (16)	9 (28.1)	3 (18.8)	4 (22.2)	0.861
Both choice hospitals, No. (%)	81 (51.3)	2 (28.6)	8 (53.3)	25 (55.6)	14 (56)	15 (46.9)	9 (56.3)	8 (44.4)	
Coiling-first hospitals, No. (%)	40 (25.3)	1 (14.3)	4 (26.7)	10 (22.2)	7 (28)	8 (25)	4 (25)	6 (33.3)	
Fasudil, No. (%)	150 (100)	7 (100)	14 (100)	44 (100)	23 (100)	31 (100)	13 (100)	18 (100)	-
Cilostazol, No. (%)	86 (57)	4 (66.7)	8 (61.5)	24 (53.3)	14 (58.3)	18 (56.3)	7 (50)	11 (64.7)	0.972
Ozagrel, No. (%)	83 (55.3)	3 (42.9)	9 (64.3)	31 (70.5)	12 (52.2)	14 (45.2)	3 (23.1)	11 (61.1)	0.060
Statin, No. (%)	49 (32.5)	0 (0)	6 (46.2)	14 (31.1)	7 (29.2)	13 (40.6)	3 (21.4)	6 (35.3)	0.427
Nicardipine, No. (%)	37 (24.5)	1 (16.7)	2 (15.4)	17 (37.8)	3 (12.5)	9 (28.1)	3 (21.4)	2 (11.8)	0.189
EPA, No. (%)	16 (10.6)	0 (0)	3 (23.1)	2 (4.4)	5 (20.8)	4 (12.5)	2 (14.3)	0 (0)	0.141
Edaravon, No. (%)	25 (16.6)	0 (0)	3 (23.1)	3 (6.7)	5 (20.8)	9 (28.1)	1 (7.1)	4 (23.5)	0.138
Clazosentan, No. (%)	7 (4.6)	0 (0)	1 (7.7)	2 (4.4)	2 (8.3)	0 (0)	1 (7.1)	1 (5.9)	0.793
Steroid, No. (%)	5 (3.3)	0 (0)	1 (7.7)	0 (0)	1 (4.2)	2 (6.3)	0 (0)	1 (5.9)	0.640
CSF drainage, No. (%)	134 (90.5)	5 (100)	14 (100)	37 (84.1)	19 (79.2)	29 (96.7)	14 (93.3)	16 (100)	0.092
IVR/DCI, Mean ± SD	1.7 ± 2.4	1.6 ± 2.1	1.2 ± 2.1	1.7 ± 2.7	1.4 ± 2.4	2 ± 2.4	2.2 ± 2.8	1.1 ± 1.4	0.610
IVR by fasdil, No. (%)	14 (9.1)	0 (0)	3 (20)	7 (15.6)	2 (8.3)	1 (3.2)	1 (6.7)	0 (0)	0.455
IVR by balloon, No. (%)	134 (87)	6 (85.7)	12 (80)	36 (80)	21 (87.5)	29 (93.5)	13 (86.7)	17 (100)	
IVR by papaverine, No. (%)	6 (3.9)	1 (14.3)	0 (0)	2 (4.4)	1 (4.2)	1 (3.2)	1 (6.7)	0 (0)	

P, Trend probability value among seven regions.

SD, Standard deviation.

CSF, cerebrospinal fluid.

DCI, delayed cerebral infarction.

EPA, eicosapentaenoic acid.

IVR, interventional radiology.

SAH, subarachnoid hemorrhage.

### 3.2. Comparison between hospitals with coil treatment priority or clip treatment priority

Among the 638 patients treated at the 37 clipping-first hospitals, 74 suffered DCI (11.6%), whereas 56 of 840 (6.7%) treated at the coiling-first hospitals suffered DCI, as shown in Table 2. In the order of clipping-first, both-choice, and coiling-first hospitals, there was a marked downward trend in the DCI frequency after coil embolization (14.7, 7.5, and 4.7%, respectively); conversely, there was an upward trend in the DCI frequency after clipping (10.9, 11.5, and 16.2%, respectively).

**Table 2:** Odds ratio (OD) and 95% confidential interval (95%CI) for incidence of delayed cerebral infarction (DCI).

	**Number (%)**	**Incidence of DCI**	**OR (95% CI)**	***P***
Hospital with < 15 SAHs	72 (45.6)	10.8 ± 17.7	Reference	
Hospital with 15 or more SAHs	86 (54.4)	9.5 ± 8.6	0.273 (0.008–9.58)	0.548
Hospital with < 16 coiling	78 (49.4)	11.9 ± 17.3	Reference	
Hospital with 16 or more coiling	80 (50.6)	8.4 ± 8.1	0.03 (0.001–1)	0.100
Hospital with < 12 clipping	78 (49.4)	8.4 ± 11.0	Reference	
Hospital with 12 or more clipping	80 (50.6)	11.7 ± 15.5	29.2 (0.863–989)	0.115
Hospital of clipping first	37 (23.4)	13.9 ± 20.4	1.15 (0.56–2.36)	0.757
Hospital of both clipping and coiling	81 (51.3)	9.6 ± 11.0	Reference	
Hospital of coiling first	40 (25.3)	7.5 ± 9.1	0.63 (0.33–1.19)	0.230
Hospital in Hokkaido	7 (4.4)	5.1 ± 5.7	0.46 (0.13–1.63)	0.313
Hospital in Tohoku	15 (9.5)	8.6 ± 9.8	1.17 (0.45–3.05)	0.790
Hospital in Kanto	45 (28.5)	9.4 ± 10.9	Reference	
Hospital in Chubu	25 (15.8)	7.8 ± 9.0	0.61 (0.29–1.28)	0.272
Hospital in Kinki	32 (20.3)	14.8 ± 16.5	1.42 (0.65–3.09)	0.465
Chugoku/Shikoku	16 (10.1)	13.9 ± 24.8	1.93 (0.68–5.53)	0.301
Kyushu	18 (11.4)	7.4 ± 6.4	0.72 (0.28–1.85)	0.564
No fasudil use	0	-	Reference	
Fasudil use	150 (100)	10.3 ± 13.6	–	-
No cilostazol use	65 (43)	14.0 ± 17.6	Reference	
Cilostazol use	86 (57)	7.8 ± 9.0	0.48 (0.27–0.82)	0.026
No ozagrel use	67 (44.7	10.0 ± 14.7	Reference	
Ozagrel use	83 (55.3)	10.7 ± 12.7	1.11 (0.63–1.94)	0.761
No statin use	102 (67.5)	11.6 ± 15.2	Reference	
Statin use	49 (32.5)	8.1 ± 9.5	0.72 (0.4–1.3)	0.359
No nicardipine use	114 (75.5)	9.8 ± 11.9	Reference	
Nicardipine use	37 (24.5)	12.6 ± 18.1	0.88 (0.46–1.67)	0.741
No edaravon use	126 (83.4)	10.0 ± 12.9	Reference	
Edaravon use	25 (16.6)	12.6 ± 17.3	0.89 (0.42–1.88)	0.802
No EPA use	135 (89.4)	10.2 ± 11.9	Reference	
EPA use	16 (10.6)	13.0 ± 24.5	1.67 (0.68–4.09)	0.346
No clazosentan use	144 (95.4)	10.6 ± 13.8	Reference	
Clazosentan use	7 (4.6)	7.1 ± 10.6	0.64 (0.17–2.38)	0.576
No steroid use	146 (96.7)	10.4 ± 13.8	Reference	
Steroid use	5 (3.3)	11.7 ± 12.5	1.16 (0.25–5.44)	0.872
No CSF drainage	14 (9.5)	13.5 ± 20.2	Reference	
CSF drainage	134 (90.5)	9.9 ± 12.9	0.61 (0.23–1.56)	0.384

P, Probability value for the logistic regression analysis.

CSF, cerebrospinal fluid.

EPA, eicosapentaenoic acid.

SAH, subarachnoid hemorrhage.

### 3.3. Investigation of preventive effect on DCI-related cerebral vasospasm

There were no significant differences in either the frequency of prophylactic drugs use or the coiling ratio among Japanese regions. The ORs and 95%CIs for the incidence of DCI are shown in Table 3. Among the 9 prophylactic drugs for preventing DCI-related cerebral vasospasm, only cilostazol was associated with significantly lower DCI frequency (OR, 0.48; 95%CIs, 0.27–0.82; P, 0.026). Since the majority responded that they used multiple prophylactic drugs, possible combinations of multiple prophylactic drugs were also investigated. The combinations of multiple prophylactic agents that were more effective in reducing DCI-related cerebral vasospasm than cilostazol alone are presented in Table 4. The most effective combination of multiple prophylactic drugs in reducing DCI related to cerebral vasospasm was cilostazol, fasudil, and statin (0.38; 0.22–0.67; 0.005). None of the multiple drug combinations, when excluding cilostazol, exhibited a preventive effect against DCI (Supplementary Table). The other questions on the survey, such as number of SAH treatments, coiling ratio, geographic region, and presence of drain placement, showed no significant association with the frequency of DCI. The efficacy of cilostazol in reducing DCI was assessed by adjustment for clipping, coiling, coiling ratio, region, and the number of SAH (Table 5). Even with various adjustments, cilostazol's effect in attenuating DCI caused by cerebral vasospasm was unchanged (OR, 0.43–0.50).

**Table 3:** Clinical characteristics of cases with different treatment strategies.

	**Total**	**Clipping first**	**Both choice**	**Coiling first**	***P***
Hospitals, No.	158	37	81	40	
SAHs, No.	3,093	638	1,615	840	0.306
Clipping, No.	1,401	522	737	142	< 0.001
Coiling, No.	1,692	116	878	698	< 0.001
DCI/SAHs, No. (%)	281 (9.1)	74 (11.6)	151 (9.3)	56 (6.7)	0.609
DCI/SAHs treated by clipping, No. (%)	165 (11.8)	57 (10.9)	85 (11.5)	23 (16.2)	0.041
DCI/SAHs treated by coiling, No. (%)	116 (6.9)	17 (14.7)	66 (7.5)	33 (4.7)	0.114
SAHs/Hospitals, mean ± SD	19.6 ± 15.1	17.2 ± 13.7	19.9 ± 16	21.0 ± 14.5	0.306
DCI/Hospitals, mean ± SD	1.8 ± 2.0	2.0 ± 2.2	1.9 ± 2.2	1.4 ± 1.4	0.609
DCI/SAHs, mean ± SD	10.1 ± 13.5	13.9 ± 20.4	9.6 ± 11.0	7.5 ± 9.1	0.241
DCI/SAHs treated by clipping, mean ± SD	1.0 ± 1.5	1.5 ± 2.1	1.0 ± 1.4	0.6 ± 0.8	0.041
DCI/SAHs treated by coiling, mean ± SD	0.7 ± 1.0	0.5 ± 0.8	0.8 ± 1.1	0.8 ± 1.0	0.114
Hospitals with < 15 SAHs, No. (%)	72 (45.6)	22 (59.5)	35 (43.2)	15 (37.5)	0.128
Hospitals with ≤ 15 SAHs, No. (%)	86 (54.4)	15 (40.5)	46 (56.8)	25 (62.5)	
Fasudil, No. (%)	150 (100)	7 (100)	14 (100)	44 (100)	-
Cilostazol, No. (%)	86 (57)	17 (50)	46 (57.5)	23 (62.2)	0.580
Ozagrel, No. (%).	83 (55.3)	21 (56.8)	47 (59.5)	15 (44.1)	0.314
Statin, No. (%).	49 (32.5)	11 (32.4)	25 (31.3)	13 (35.1)	0.916
Nicardipine, No. (%).	37 (24.5)	11 (32.4)	20 (25)	6 (16.2)	0.284
Edaravon, No. (%).	25 (16.6)	6 (17.6)	13 (16.3)	6 (16.2)	0.981
EPA, No. (%).	16 (10.6)	4 (11.8)	8 (10)	4 (10.8)	0.960
Clazosentan, No. (%).	7 (4.6)	2 (5.9)	3 (3.8)	2 (5.4)	0.856
Steroid, No. (%).	5 (3.3)	2 (5.9)	3 (3.8)	0 (0)	0.365
CSF drainage, No. (%).	134 (90.5)	25 (86.2)	73 (90.1)	36 (94.7)	0.488
IVR/DCI, mean ± SD	1.7 ± 2.4	1.9 ± 2.6	1.6 ± 2.1	1.6 ± 2.6	0.973
IVR by fasdil, No. (%).	14 (9.1)	1 (2.9)	8 (9.9)	5 (12.8)	0.591
IVR by balloon, No. (%).	134 (87)	32 (94.1)	69 (85.2)	33 (84.6)	
IVR by papaverine, No. (%).	6 (3.9)	1 (2.9)	4 (4.9)	1 (2.6)	

P, Probability value for the Kruskal-Wallis test and Chi-square test.

SD, Standard deviation.

CSF, cerebrospinal fluid.

DCI, delayed cerebral infarctions.

EPA, eicosapentaenoic acid.

IVR, interventional radiology.

SAH, subarachnoid hemorrhage.

**Table 4:** Efficacy of combinations of multiple prophylactic drugs in reducing delayed cerebral infarction (DCI) due to cerebral vasospasm.

	**OR (95% CI)**	***P***
Only cilostazol	0.48 (0.27–0.82)	0.026
Cilostazol + Fasudil + Ozagrel	0.43 (0.22–0.86)	0.046
Cilostazol + Fasudil + Edaravon	0.40 (0.22–0.71)	0.008
Cilostazol + Fasudil + Statin	0.38 (0.22–0.67)	0.005
Cilostazol + Fasudil + Edaravon + Statin	0.43 (0.24–0.74)	0.012
Cilostazol + Fasudil + Edaravon + Nicardipine	0.48 (0.27–0.83)	0.028

OR, odds ratio.

CI, confidential interval.

P, Probability value for the logistic regression analysis.

**Table 5:** Efficacy of cilostazol in reducing delayed cerebral infarction (DCI) due to cerebral vasospasm.

**Adjusted**	**OR (95% CI)**	***P***
No adjustment	0.48 (0.27–0.82)	0.026
SAH number	0.46 (0.27–0.76)	0.012
Number of clipping	0.43 (0.26–0.71)	0.006
Number of coiling	0.48 (0.28–0.82)	0.024
Coiling ratio	0.49 (0.29–0.86)	0.035
Region	0.50 (0.29–0.86)	0.036

OR, odds ratio.

CI, confidential interval.

P, Probability value for the logistic regression analysis.

SAH, subarachnoid hemorrhage.

## 4. Discussion

This study revealed the incidence of DCI associated with cerebral vasospasm after aneurysmal SAH and trends in prophylactic drugs throughout Japan in 2021. The incidence of DCI had a median of 6.6%, and mean of 10.1% per SAH. This study confirmed that endovascular coil embolization had significantly lower incidence of DCI related to cerebral vasospasm, compared to microsurgical clipping with craniotomy. This result is in line with previous reports (10–14). In a recent systematic review of aneurysmal SAH, clipping increased the risk of vasospasm rate at discharge by 45% compared to coiling (14). Dehdashti et al. reported that symptomatic vasospasm and ischemic infarction with permanent neurological deficit occurred in 25% and 9% in the patients treated with surgical clipping and 15% and 7% in those treated with endovascular coiling, respectively (10). The definition of DCI in this study was symptomatic cerebral infarction due to vasospasm, and almost consistent with this study. In addition, our findings indicate that DCI may be less likely to occur after a familiar procedure that is routinely performed in the hospital. Specifically, DCI associated with cerebral vasospasm is less likely to occur after clipping in clipping-first hospitals, while it is less likely to occur after coiling in coiling-first hospitals. With the widespread use of coil embolization for ruptured aneurysms, DCI associated with cerebral vasospasm has been on the decline, but is still far from a level at which the DCI can be considered vanquished. The incidence of DCI outside Japan has been reported to be 11 to 47 % (1, 2, 6, 11, 15, 16) which is higher than in Japan. One reason may be that prophylactic drug use is much more limited outside Japan. Many Japanese physicians have preferred to use multiple prophylactic drugs, especially cilostazol, but it was not mentioned in the new American Heart Association/American Stroke Association guidelines for the management of patients with aneurysmal SAH in 2023 (3). Cilostazol is the most potent vasodilator among antiplatelet agents and is increasingly considered effective in cerebral vasospasm. In Japan, many hospitals use multiple prophylactic drugs, especially cilostazol, which has recently become more common. Cilostazol is the most potent vasodilator among antiplatelet agents and is increasingly considered effective against cerebral vasospasm (13, 16–24). The preventive effect of cilostazol on cerebral vasospasm has been demonstrated in several RCTs (16, 18, 20), systematic review and meta-analysis articles (19, 21–24). In addition, we found that multidrug combination with cilostazol, fasudil, and statin was the most effective in reducing DCI-related cerebral vasospasm. However, a standard protocol based on rigorously evaluated evidence should be established, because Japanese physicians had used a variety of prophylactic drugs, believing them to be effective based on personal experience. The second reason may be that Japanese neurosurgeons are generally responsible for managing SAH patients from admission to discharge. They have a strong desire to ensure that the patients they operated on do well and go home.

Several limitations of this study should be considered. The first is that detailed information of each SAH patient, including the severity of SAH or management of cerebral vasospasm, was not available, but only hospital-specific information. DCI caused by cerebral vasospasm is known to be strongly associated with the severity of SAH. This questionnaire only surveyed trends of management of cerebral vasospasm and the incidence of DCI at each hospital. The second limitation was the low survey collection rate of approximately 30%. This could indicate possible sampling bias. However, the distribution of registered SAH cases in each region was very similar to the national population ratio in Japan. Next, however, we would like to directly utilize data from the Japanese Stroke Association Annual Clinical Status Survey to investigate the changes in prophylactic drugs use and frequency of DCI as a longitudinal study. Finally, modalities for detecting DCI and methods of DCI may vary from hospital to hospital. Indeed, regional disparities in the incidence of DCI were thought to possibly include this bias. Specifically, only the Hokkaido region responded that microsurgical clipping had a lower incidence of DCI rather than coil embolization, but some clipping-first hospitals in the Hokkaido region routinely asserted that the microsurgical removal of as much subarachnoid hematoma as possible in SAH helps to prevent cerebral vasospasm (25, 26). However, previous systematic review and meta-analysis reports support the view that microsurgical manipulation accelerates cerebral vasospasm after SAH (16, 19, 21–24).

## 5. Conclusions

Based on a hospital-based nationwide survey of the managements for aneurysmal SAH including DCI in Japan, we identified trends in prophylactic drugs to prevent cerebral vasospasm after aneurysmal SAH in 2021. Endovascular coil embolization of cerebral aneurysms and cilostazol administration alone or in combination with fasudil and statin proved effective in reducing DCI related to cerebral vasospasm in 2021. Treatment modalities for SAH are evolving daily, and the best treatment should be selected based on the latest knowledge and with full consideration of the relative risks and benefits to the patients treated with aneurysmal SAH, rather than on the experience of individual clinicians. Furthermore, we believe that a comparative analysis with data from other countries would provide valuable insights into international differences in SAH management and the effectiveness of prophylactic treatments for DCI associated with cerebral vasospasm.

## Data availability statement

The original contributions presented in the study are included in the article/Supplementary material, further inquiries can be directed to the corresponding author.

## Ethics statement

Ethical approval was not required for the study involving humans in accordance with the local legislation and institutional requirements. Written informed consent to participate in this study was not required from the participants or the participants' legal guardians/next of kin in accordance with the national legislation and the institutional requirements.

## Author contributions

YN: Writing—original draft, Conceptualization, Data curation, Investigation, Methodology, Project administration, Resources, Validation, Visualization. SY: Formal analysis, Validation, Writing—original draft. MU: Data curation, Writing—review and editing. TY: Data curation, Writing—review and editing. YH: Data curation, Writing—review and editing. HK: Supervision, Writing—review and editing. MT: Supervision, Writing—review and editing. TI: Supervision, Writing—review and editing. KI: Supervision, Writing—review and editing. MMo: Supervision, Writing—review and editing. MMa: Supervision, Writing—review and editing.
