# Supplementary material for: Japanese nationwide questionnaire survey on delayed cerebral infarction due to vasospasm after subarachnoid hemorrhage
Source: Front Neurol. 2023 Nov 2;14:1296995. doi: 10.3389/fneur.2023.1296995 (PMC10654625; doi:10.3389/fneur.2023.1296995)
Supplement: Supplementary file 1 [file Table_1.docx]

Supplementary Table

|  | OR (95% CI) | *P* |
| --- | --- | --- |
| Fasudil + Ozagrel | 1.25 (0.36–4.30) | 0.770 |
| Ozagrel + Edaravon | 1.29 (0.68–2.43) | 0.507 |
| Ozagrel + Statin | 1.17 (0.65–2.10) | 0.660 |
| Edaravon + Statin | 0.98 (0.54–1.76) | 0.952 |
| Fasudil + Edaravon | 0.94 (0.34–2.65) | 0.927 |
| Fasudil + Statin | 1.39 (0.43–4.51) | 0.646 |
| Nicardipine + Ozagrel | 1.07 (0.58–1.98) | 0.847 |
| Nicardipine + Edaravon | 0.79 (0.43–1.44) | 0.521 |
| Nicardipine + Statin | 0.77 (0.43–1.35) | 0.438 |
| Fasudil + Ozagrel + Edaravon | 0.87 (0.49–1.54) | 0.685 |
| Fasudil + Ozagrel + Statin | 0.85 (0.46–1.57) | 0.660 |
| Fasudil + Ozagrel + Nicardipine | 1.02 (0.58–1.82) | 0.946 |
| Fasudil + Edaravon + Statin | 0.64 (0.36–1.14) | 0.205 |
| Fasudil + Edaravon + Nicardipine | 0.91 (0.50–1.65) | 0.799 |
| Fasudil + Nicardipine + Statin | 0.67 (0.38–1.18) | 0.249 |
| Ozagrel + Edaravon + Statin | 0.94 (0.47–1.86) | 0.874 |
| Ozagrel + Edaravon + Nicardipine | 0.88 (0.45–1.72) | 0.751 |
| Edaravon + Statin + Nicardipine | 0.59 (0.26–1.33) | 0.289 |
| Fasudil + Ozagrel + Edaravon + Statin | 0.93 (0.53–1.65) | 0.846 |
| Fasudil + Ozagrel + Edaravon + Nicardipine | 1.14 (0.64–2.04) | 0.706 |
| Fasudil + Ozagrel + Edaravon + Statin + Nicardipine | 0.76 (0.41–1.39) | 0.455 |
