# Supplementary material for: Japanese nationwide questionnaire survey on delayed cerebral infarction due to vasospasm after subarachnoid hemorrhage
Source: Front Neurol. 2023 Nov 2;14:1296995. doi: 10.3389/fneur.2023.1296995 (PMC10654625; doi:10.3389/fneur.2023.1296995)
Supplement: Supplementary file 2 [file Data_Sheet_1.docx]

**Request for your cooperation in a survey on the treatment and management of subarachnoid hemorrhage due to ruptured cerebral aneurysm**

　　　　　　　　　　　　　　　　　　　　　　　　　 November 2022

　With the cooperation of the Japanese Society for Surgery of Stroke, we would like to conduct a questionnaire survey on "treatment and management of subarachnoid hemorrhage (SAH) due to ruptured cerebral aneurysm" to the hospitals in where technical supervisors and technical certifiers of the Japanese Society on Surgery for Cerebral Stroke work.

　As described in the Japanese guidelines for the management of Stroke, there is no standard method (unified protocol) for the use of prophylactic drugs to prevent cerebral vasospasm, and each hospital or each doctor has its original methods. Under such circumstances, the postoperative managements of SAH will be change dramatically in the future with clazosentan approval by the Japanese insurance system in April 2022.

At the 39th SAH/Spasm Symposium, a symposium will be held to discuss the differences before and after clazosentan approval. The purpose of this survey is to clarify the actual situation regarding the managements and frequency of cerebral vasospasm after SAH prior to the clazosentan usage.

　The survey does not require any personal information of the patients with SAH and hospitals, and collect only the number of patients treated with SAH and the name of the region where the hospital was located. Therefore, there is no need to approval by each ethics committee of your hospital. In addition, if your hospital has more than one technical supervisors and technical certifiers of the Japanese Society on Surgery for Cerebral Stroke, please answer this survey by only one doctor as a representative of your hospital. The survey will be conducted using Google form. Please fill out the survey form by clicking on the URL or QR code below. We apologize for the inconvenience, but we would appreciate your response by November 25. Please note that this survey form is always secured by SSL, and is also encrypted with multiple layers of encryption.

※ URL https://forms.gle/k9GHmoouUmqhnZPD8


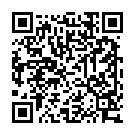
QR Code

**[Principal Investigator]**

Professor Mitsuhito Mase,

Department of Neurosurgery, Nagoya City University Hospital

**[Research Collaboration]**

SAH/Spasm Symposium Society

**[Inquiries]**

Doctor Yusuke Nishikawa

Assistant Professor, Department of Neurosurgery, Nagoya City University

1 Kawasumi, Mizuho-cho, Mizuho-ku, Nagoya 052-853-8286

**Questionnaire for the treatment and management of SAH due to ruptured cerebral aneurysm**

This survey is a questionnaire for the purpose of investigating the number of patients diagnosed with SAH due to cerebral aneurysm rupture at your hospital and receiving treatment for cerebral aneurysm to prevent rerupture and the type of treatments for cerebral vasospasm. Therefore, please answer the following questions about SAH patients who received treatment to prevent re-rupture of the cerebral aneurysm within 72 hours after onset and to prevent cerebral vasospasm at your hospital from January 1, 2021 to December 31, 2021.

Question1. Which branch of the Japanese Neurosurgical Association does your institution belong to?

⃞Hokkaido, ⃞Tohoku, ⃞Kanto, ⃞Chubu, ⃞Kinki, ⃞Chugoku-Shikoku, ⃞Kyushu

Question2-1. How many patients treated with ruptured cerebral aneurysms within 72 hours from onset to prevent re-rupture during the following period; January 1, 2021 to December 31, 2021 at your hospital?

Answer 2-1. ___ people

Question2-2. How many patients were treated with clipping (open surgery) among SAH patients answered in Question 2-1?

Answer 2-2. ___ people

Question 2-3. How many patients were treated with coil embolization (endovascular surgery) among SAH patients answered in Question 2-1?

Answer 2-3. ___ people

Question 4. How many patients were treated with both clipping and coiling among SAH patients answered in Question 2-1?

Answer 4. ___ people

Question5. Are there any institutional standards (protocols) for postoperative management, prevention and treatment for cerebral vasospasm (prophylactic drugs, cerebrospinal fluid (CSF) drainage methods, examination to confirm cerebral vasospasm, etc.)?

Answer 5. ⃞ Yes (90% or more)

⃞ Although there is a institutional standard (75-90%), some of them are left to their own doctor (original management).

⃞ Although there is a institutional standard (50-75%), it is basically left to their own doctor (original management).

⃞ No. There is no institutional standard. All managements are left to their own doctor.

The next question is about drugs, which do not include blood products or blood transfusions.

Question 6-1. Please check all applicable drugs that were used in principle (in about 80% or more of the patients) during the first 14 days after the SAH onset in the patients who were treated with clipping (open surgery). (multiple answers allowed)

Answer 6-1. ⃞ Fasudil hydrochloride, ⃞ Ozagrel sodium, ⃞Cilostazol, ⃞ Edaravone,

⃞ Statins, ⃞ Steroids, ⃞ Nicardipine (not for antihypertensive),

⃞ Clazosentan, ⃞ EPA, ⃞ Other

Question 6-2. If you answered "other" in Question 6-1, please provide details. ( )

Question 7-1. Please check all applicable drugs that were used in principle (in about 80% or more of patients) during the first 14 days after the SAH onset in the patients who were treated with coiling (endovascular surgery). (multiple answers allowed)

Answer 7-1. ⃞ Fasudil hydrochloride, ⃞ Ozagrel sodium, ⃞Cilostazol, ⃞ Edaravone,

⃞ Statins, ⃞ Steroids, ⃞ Nicardipine (not for antihypertensive),

⃞ Clazosentan, ⃞ EPA, ⃞ Other

If you answered "other" in Question 7-1, please provide details. ( )

Question 8-1. Please check all applicable drugs that were used in the majority (approximately 50-80% of patients) during the first 14 days after the SAH onset in the patients who were treated with clipping (open surgery). (multiple answers allowed)

Answer 8-1. ⃞ Fasudil hydrochloride, ⃞ Ozagrel sodium, ⃞Cilostazol, ⃞ Edaravone,

⃞ Statins, ⃞ Steroids, ⃞ Nicardipine (not for antihypertensive),

⃞ Clazosentan, ⃞ EPA, ⃞ Other

Question 8-2. If you answered "other" in Question 8-1, please provide details. ( )

Question 9-1. Please check all applicable drugs that were used in the majority (approximately 50-80% of patients) during the first 14 days after the SAH onset in the patients who were treated with coiling (endovascular surgery). (multiple answers allowed)

Answer 9-1. ⃞ Fasudil hydrochloride, ⃞ Ozagrel sodium, ⃞Cilostazol, ⃞ Edaravone,

⃞ Statins, ⃞ Steroids, ⃞ Nicardipine (not for antihypertensive),

⃞ Clazosentan, ⃞ EPA, ⃞ Other

Question 9-2. If you answered "other" in Question 9-1, please provide details. ( )

Question 10. Is there any difference in the drugs used in SAH patients with severe symptoms (WFNS grade IV and V) compared to those with mild symptoms?

Answer 10. ⃞ Yes ⃞ No

Question 11-1. This question is for institutions that answered "Yes" to Question 10.

Please check all applicable drugs that were used in principle (in approximately 80% or more of patients) during the first 14 days after the SAH onset in the patients who were treated with clipping (open surgery). (multiple answers allowed)

Answer 11-1. ⃞ Fasudil hydrochloride, ⃞ Ozagrel sodium, ⃞Cilostazol, ⃞ Edaravone,

⃞ Statins, ⃞ Steroids, ⃞ Nicardipine (not for antihypertensive),

⃞ Clazosentan, ⃞ EPA, ⃞ Other

If you answered "other" in Question 11-1, please provide details. ( )

Question 12-1. This question is for institutions that answered "Yes" to Question 10.

Please check all applicable drugs that were used in principle (in approximately 80% or more of patients) during the first 14 days after the SAH onset in the patients who were treated with coiling (endovascular surgery). (multiple answers allowed)

Answer 12-1. ⃞ Fasudil hydrochloride, ⃞ Ozagrel sodium, ⃞Cilostazol, ⃞ Edaravone,

⃞ Statins, ⃞ Steroids, ⃞ Nicardipine (not for antihypertensive),

⃞ Clazosentan, ⃞ EPA, ⃞ Other

Question 12-2. If you answered "other" in Question 12-1, please provide details. ( )

Next, I would like to ask about CSF drainage for patients diagnosed with Fisher group 3 SAH on CT scan.

Question 13. Does your hospital use irrigation therapy* as one of the CSF drainage methods?

*Using CSF drainage catheters for the purposes other than CSF drainage, such as intrathecal injection of some drugs (for example, urokinase or tissue-type plasminogen activator).

Answer 13. ⃞ Yes ⃞ No

Question 14. Please check the CSF drainage methods performed in majority of SAH patients treated with clipping.

Answer 14. ⃞ Spinal drainage, ⃞ Cisternal drainage, ⃞ Spinal and Cisternal drainage,

⃞ Spinal and Ventricular drainage, ⃞ Cisternal and Ventricular drainage,

⃞ Ventricular drainage only, ⃞ Not performed drainage in principle

Question 15. Please check the CSF drainage methods performed in majority of SAH patients treated with coiling.

Answer 15. ⃞ Spinal drainage, ⃞ Cisternal drainage, ⃞ Spinal and Cisternal drainage,

⃞ Spinal and Ventricular drainage, ⃞ Cisternal and Ventricular drainage,

⃞ Ventricular drainage only, ⃞ Not performed drainage in principle

We would like to ask you about examinations and endovascular treatments (e.g., selective arterial infusion therapy) performed during the postoperative vasospasm periods.

Question 16. Did you measure central venous pressure (CVP)?

Answer 16. ⃞ Yes ⃞ No

Question 17. Approximately how many times for blood tests (e.g., blood count, biochemistry) were done in your hospital during the first 14 days after the SAH onset?

Answer 17. ⃞ 0-4 times, ⃞ 5-8 times, ⃞ 9-11 times, ⃞ 12 or more times

Question 18-1. Please select all applicable examinations that are performed to detect cerebral vasospasm (examinations performed at certain times during the period of cerebral vasospasm). (multiple answers allowed)

Answer 18-1. ⃞ Transcranial doppler, ⃞ MRA, ⃞ CT angiography, ⃞ DSA, ⃞ MR perfusion, ⃞ CT perfusion, ⃞ SPECT, ⃞ EEG, ⃞ NIRO, ⃞ Other ( )

Question 18-2. If you answered "other" in Question 18-1, please provide details. ( )

We would like to ask about the treatments for cerebral vasospasm.

Question 19-1. please check all additional treatments for cerebral vasospasm. (multiple answers allowed)

Answer 19-1. ⃞ Triple H therapy, ⃞ Hyperdynamic therapy, ⃞ Anticonvulsants,

⃞ Endovascular therapy, ⃞ Antiplatelet therapy (such as ozagrel), ⃞ Other

Question 19-2. If you answered "other" in Question 19-1, please provide details. ( )

Question 19-3. Please check the criteria for endovascular treatment for cerebral vasospasm.

Answer 19-3.

⃞ Both symptomatic and cerebral vasospasm confirmed by DSA

⃞ Cerebral vasospasm confirmed by DSA, regardless of symptoms

⃞ Other

Question 19-4. If you answered "other" in Question 19-3, please provide details. ( )

Question 19-5. Which endovascular treatment for cerebral vasospasm is your first choice?

Answer 19-5. ⃞ Fasudil hydrochloride infusion therapy, ⃞ Papaverine hydrochloride infusion therapy, ⃞ Percutaneous angioplasty

Question 20. How many patients were treated with endovascular therapy for cerebral vasospasm? (If more than one endovascular treatment was performed in one patient, please specify one people.)

Answer 20. ___ people

Question 21-1. How many patients were symptomatic cerebral infarctions (new cerebral infarction on imaging) caused by cerebral vasospasm?

Answer 21-1. ___ people

Question 21-2. How many patients were treated with clipping (open surgery) among SAH patients diagnosed with symptomatic cerebral infarctions you answered in Question 21-1?

Answer 21-2. ___ people

Question 21-3. How many were treated with coiling (endovascular surgery) among SAH patients diagnosed with symptomatic cerebral infarctions you answered in Question 21-1?

Answer 21-3. ___ people

Question 21-4. How many were treated with both clipping and coiling among SAH patients diagnosed with symptomatic cerebral infarctions you answered in Question 21-1?

Answer 21-4. ___ people

Thank you for your time and cooperation.

We will be featuring these results at the SAH/Spasm Symposium at Stroke2023.

**[Principal Investigator]**

Professor Mitsuhito Mase,

Department of Neurosurgery, Nagoya City University Hospital

**[Research Collaboration]**

SAH/Spasm Symposium Society

**[Inquiries]**

Doctor Yusuke Nishikawa

Assistant Professor, Department of Neurosurgery, Nagoya City University

1 Kawasumi, Mizuho-cho, Mizuho-ku, Nagoya 052-853-8286
