# Supplementary material for: Japanese nationwide questionnaire survey on delayed cerebral infarction due to vasospasm after subarachnoid hemorrhage
Source: Front Neurol. 2023 Nov 2;14:1296995. doi: 10.3389/fneur.2023.1296995 (PMC10654625; doi:10.3389/fneur.2023.1296995)
Supplement: Supplementary file 3 [file Data_Sheet_2.docx]

破裂脳動脈瘤によるくも膜下出血の治療・管理に関する調査へのご協力のお願い

　　　　　　　　　　　　　　　　　　　　　　　　　 2022年11月

　この度、脳卒中の外科学会のご協力の下、「破裂脳動脈瘤によるくも膜下出血の治療・管理」について日本脳卒中の外科学会技術指導医、技術認定医の在籍施設を対象にアンケート調査を行わせて頂きたく存じます。

　脳血管攣縮の予防や治療のための使用薬剤や管理方法はガイドラインにも様々な記載があるように統一された方法はなく、各施設で様々な工夫が行われています。そのような中、2022年4月よりclazosentanが保険診療で承認されたことで、今後くも膜下出血の術後管理は大きく変わっていくことが予想されます。第39回SAH/スパズム・シンポジウムではclazosentan使用前後での違いについてシンポジウムで取り上げる予定にしています。本調査研究では、clazosentan導入前までのくも膜下出血後脳血管攣縮の予防や治療、管理方法に関する実態を明らかにすることを目的としております。

　今回の調査では個人情報を記入する部分はなく、施設としての治療方針と件数をご回答いただく内容になっています。このため倫理委員会等を通していただく必要はありません。また、脳卒中の外科学会技術指導医、技術認定医が複数在籍されている施設では、代表してどなたかお一人から回答いただけますと幸いです。調査方法はGoogle formを用いたアンケート調査を行わせていただきます。下部のURLもしくはQRコードから調査票にご回答ください。大変お手数をおかけしますが、11月25日までにご回答頂ければ幸いです。なお，この調査票は常時SSL化されているのに加え、何重にも暗号をかけ、厳重にセキュリティ管理されています。

- URL 　　https://forms.gle/k9GHmoouUmqhnZPD8

QRコード
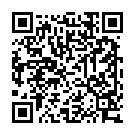


【研究代表】

名古屋市立大学病院　脳神経外科　教授　間瀬光人

【研究協力】

SAH/スパズム・シンポジウム学会

【お問い合わせ】

名古屋市立大学　脳神経外科　助教　西川祐介

　名古屋市瑞穂区瑞穂町川澄１　　052-853-8286

破裂脳動脈瘤によるくも膜下出血の治療・管理に関する調査票

本調査は、脳動脈瘤破裂によるくも膜下出血と診断され、脳動脈瘤について再破裂予防のための治療を行った症例を対象としたアンケート調査です。このため対象は2021年1月1日から2021年12月31日までの期間で発症から72時間以内に破裂脳動脈瘤に対して再破裂予防の治療を行った症例とします。

質問1. 貴施設は日本脳神経外科学会の支部会でどこの所属になりますか？

□北海道支部、□東北支部、□関東支部、□中部支部、□近畿支部、□中国四国支部、□九州支部

質問2-1. 貴施設では対象期間(2021年1月1日から2021年12月31日まで)に発症から72時間以内に破裂脳動脈瘤に対して再破裂予防の治療を行った症例は何例ありますか？

解答2-1. ______例

質問2-2. 質問2-1.で回答した症例の治療で開頭手術は何例ありますか？

解答2-2.　＿＿＿例

質問2-3. 質問2-1.で回答した症例の治療で脳血管内治療は何例ありますか？

解答2-3.　＿＿＿例

質問4. 質問1.で回答した症例の治療で開頭手術+脳血管内治療は何例ありますか？

解答4.　＿＿＿例

質問5．術後の管理，脳血管攣縮の予防・治療（薬剤、髄液ドレナージ法、検査など）に施設としての基準（プロトコル）はありますか？

解答5. □はい（90%以上）

□おおむねの基準（75-90%）はあるが一部は担当医（主治医）に一任している

□一部の基準(50-75%)はあるが、基本的には担当医（主治医）に一任している

□ない。各担当医（主治医）に一任している

次に薬剤について質問します。ここでいう薬剤には血液製剤や輸血は含まれません。

質問6-1. 開頭手術を行った症例で、発症から14日間までの間に原則使用した（約80%以上の症例で使用した）薬剤を該当するものすべてチェックしてください。

解答6-1. □塩酸ファスジル、□オザグレルナトリウム、□シロスタゾール、□エダラボン、

　　　　□スタチン、□ステロイド、□ニカルジピン（降圧目的以外）、□クラゾセンタン、

　　　　□EPA製剤、□その他

質問6-2. 質問6-1でその他とお答えになった方は内容をご記入ください

質問7-1. 血管内治療を行った症例で、発症から14日間までの間に原則使用した（約80%以上の症例で使用した）薬剤を該当するものすべてチェックしてください。

解答7-1. □塩酸ファスジル、□オザグレルナトリウム、□シロスタゾール、□エダラボン、

　　　　□スタチン、□ステロイド、□ニカルジピン（降圧目的以外）、□クラゾセンタン、

　　　　□EPA製剤、□その他

質問7-2. 質問7-1でその他とお答えになった方は内容をご記入ください

質問8-1.　開頭手術を行った症例で、発症から14日間までの間に使用した症例が多い（約50-80%の症例で使用した）薬剤を該当するものすべてチェックしてください。

解答8-1. □塩酸ファスジル、□オザグレルナトリウム、□シロスタゾール、□エダラボン、

　　　　□スタチン、□ステロイド、□ニカルジピン（降圧目的以外）、□クラゾセンタン、

　　　　□EPA製剤、□その他

質問8-2. 質問8-1でその他とお答えになった方は内容をご記入ください

質問9-1. 血管内治療を行った症例で、発症から14日間までの間に使用した症例が多い（約50-80%の症例で使用した）薬剤を該当するものすべてチェックしてください。

解答9-1. □塩酸ファスジル、□オザグレルナトリウム、□シロスタゾール、□エダラボン、

　　　　□スタチン、□ステロイド、□ニカルジピン（降圧目的以外）、□クラゾセンタン、

　　　　□EPA製剤、□その他

質問9-2. 質問9-1でその他とお答えになった方は内容をご記入ください

質問10.重症例(WFNS garde Ⅳ、Ⅴ)では軽症例と比較して使用する薬剤に違いがありますか？

解答10. □はい

　　　 □いいえ

質問11-1. 質問10で「はい」と答えた施設に質問します。開頭手術を行った重症例で、発症から14日間までの間に原則使用した（約80%以上の症例で使用した）薬剤を該当するものすべてチェックしてください。

解答11-1. □塩酸ファスジル、□オザグレルナトリウム、□シロスタゾール、□エダラボン、

　　　　□スタチン、□ステロイド、□ニカルジピン（降圧目的以外）、□クラゾセンタン、

　　　　□EPA製剤、□プロポフォールなどの麻酔薬（鎮静薬）、□その他

質問11-2. 質問11-1でその他とお答えになった方は内容をご記入ください

質問12-1. 質問10で「はい」と答えた施設に質問します。血管内治療を行った重症例で、発症から14日間までの間に原則使用した（約80%以上の症例で使用した）薬剤を該当するものすべてチェックしてください。

解答12-1. □塩酸ファスジル、□オザグレルナトリウム、□シロスタゾール、□エダラボン、

　　　　□スタチン、□ステロイド、□ニカルジピン（降圧目的以外）、□クラゾセンタン、

　　　　□EPA製剤、□プロポフォールなどの麻酔薬（鎮静薬）、□その他

質問12-2. 質問12-1でその他とお答えになった方は内容をご記入ください

次にFisher group3の症例について，くも膜下出血排出を目的とした髄液ドレナージについてお聞きします。

質問13. 貴施設では髄液ドレージ法として灌流療法^※^を取り入れていますか？

※髄液ドレナージチューブから薬剤の注入など排液以外の目的で使用している

解答13. □はい

　　　　□いいえ

質問14. 開頭手術をした症例で行った髄液ドレナージ法にチェックしてください。（多くの症例で行った方法をお答えください）

解答14. □腰椎ドレナージ、□脳槽ドレナージ、□腰椎ドレナージ＋脳槽ドレナージ、

　　　　□腰椎ドレナージ＋脳室ドレナージ、□脳槽ドレナージ＋脳室ドレナージ、

　　　　□脳室ドレナージのみ、□原則行わない

質問15. 脳血管内治療をした症例で行った髄液ドレナージ法にチェックしてください。（多くの症例で行った方法をお答えください）

解答15. □腰椎ドレナージ、□脳槽ドレナージ、□腰椎ドレナージ＋脳槽ドレナージ、

　　　　□腰椎ドレナージ＋脳室ドレナージ、□脳槽ドレナージ＋脳室ドレナージ、

　　　　□脳室ドレナージのみ、□原則行わない

最後に術後から脳血管攣縮期に行った検査および脳血管内治療（選択的動注療法など）についてお聞きします。

質問16. 中心静脈圧(CVP)の測定はしていましたか？

解答16. □はい

　　　　□いいえ

質問17. 術後から14日目までで血液検査（血算、生化学）はおおむね何回行いましたか？

解答17. □0-4回、□5-8回、□9-11回、□12回以上

質問18-1. 脳血管攣縮のモニタリングとして行っている検査（脳血管攣縮期間中の決まった時期に行っている検査）にあてはまるものすべて選んでください。

解答18-1. □経頭蓋ドップラー、□MRA、□CT angiography、□脳血管撮影、□MR perfusion、□CT perfusion、□SPECT、□脳波、□NIRO、□その他( )

質問18-2. 質問18-1でその他とお答えになった方は内容をご記入ください

最後に脳血管攣縮の治療についてお聞きします。

質問19-1. 症候性脳血管攣縮に対して追加して行う治療を全て選んでください

解答19-1. □triple H療法、□hyperdynamic療法、□抗けいれん薬、□脳血管内治療、□抗血小板療法（オザグレルなど）、□その他

質問19-2. 質問19-1でその他とお答えになった方は内容をご記入ください

質問19-3. 症候性脳血管攣縮に対して脳血管内治療を行う基準をえらんでください。

解答19-3.

□症候性かつ脳血管撮影上の脳血管攣縮を認める

□症候にかかわらず脳血管撮影上の脳血管攣縮を認める

□その他

質問19-4. 質問19-3でその他とお答えになった方は内容をご記入ください。

質問19-5. 脳血管攣縮に対する血管内治療は第１選択としてどの治療をおこないますか？

解答19-5. □塩酸ファスジル動注療法、□パパベリン塩酸塩動注療法、□経皮的血管形成術

質問20. 対象症例のうち何例に脳血管攣縮に対する血管内治療を行いましたか？（１症例に複数回行った場合は１例としてください）

解答20. ＿＿＿＿例

質問21-1. 対象症例のうち脳血管攣縮が原因の症候性の脳梗塞（画像で新たな脳梗塞を認めた）は何例に発症しましたか？

解答21-1. ＿＿＿＿例

質問21-2. 質問21-1.で回答した症例の治療で開頭手術は何例ありますか？

解答21-2. ＿＿＿＿例

質問21-3. 質問21-1.で回答した症例の治療で脳血管内治療は何例ありますか？

解答21-3. ＿＿＿＿例

質問21-4. 質問21-1.で回答した症例の治療で開頭手術+脳血管内治療は何例ありますか？

解答21-4. ＿＿＿＿例

ご多忙の中、ご協力いただきありがとうございました。この結果はStroke2023のSAH/スパズム・シンポジウムで取り上げさせていただきます。

【研究代表】

名古屋市立大学病院　脳神経外科　教授　間瀬光人

【研究協力】

SAH/スパズム・シンポジウム学会

【お問い合わせ】

名古屋市立大学　脳神経外科　助教　西川祐介

　名古屋市瑞穂区瑞穂町川澄１　　052-853-8286
